# ^99m^Tc‑labeled single-domain antibody for SPECT/CT assessment of HER2 expression in diverse cancer types

**DOI:** 10.1007/s00259-022-06066-3

**Published:** 2022-12-09

**Authors:** Betül Altunay, Andreas Goedicke, Oliver H. Winz, Fabian Hertel, Dirk von Mallek, Levente K. Meszaros, Gitasha Chand, Hans-Jürgen Biersack, Elmar Stickeler, Katja Krauss, Felix M. Mottaghy

**Affiliations:** 1 Department of Nuclear Medicine, University Hospital Aachen, RWTH Aachen University, Pauwelsstr. 30, 52074 Aachen, Germany; 2 Nanomab Technology (UK) Ltd., 720 Centennial Court, Centennial Park, Elstree , WD6 3SY Hertfordshire UK; 3 Radiopharm Theranostics Ltd, 62 Lygon Street, Carlton South, Victoria, 3053 Australia; 4 Department of Nuclear Medicine, University Hospital Bonn, Sigmund-Freud-Str. 25, 53127 Bonn, Germany; 5 Department of Gynecology and Obstetrics, University Hospital RWTH Aachen, Pauwelsstr. 30, 52074 Aachen, Germany; 6 Center of Integrated Oncology (CIO), Universities of Aachen, Bonn, Cologne and Düsseldorf, Kerpener Str. 62, 50937 Cologne, Germany; 7 Department of Radiology and Nuclear Medicine, Maastricht University Medical Center (MUMC+), P. Debeylaan 25, 6202 Maastricht, The Netherlands

**Keywords:** HER2, Single domain antibody, Nanobody, Cancer, Nuclear imaging, SPECT

## Abstract

**Abstract:**

The expression status of human epidermal growth factor receptor 2 (HER2) in cancer predicts response to HER2-targeted therapy. Therefore, its accurate determination is of utmost importance. In recent years, there has been an increase in research on noninvasive techniques for molecular imaging, as this method offers the advantages of a more accurate determination of HER2 status without the need for multiple biopsies. The technetium-labeled single-domain antibody RAD201, previously known as ^99m^Tc-NM-02, has been shown to be safe for use in breast cancer imaging with reasonable radiation doses, favorable biodistribution, and imaging characteristics.

**Methods:**

A total of six HER2-positive, heavily pretreated patients with different cancer types aged between 42 and 69 years (5 women and 1 man; the median age of 55.5) have been examined. In six of seven scans, the patients were administered 500 ml of Gelofusine® solution (40 mg/ml) for radiation protection before the tracer injection (434 ± 42 MBq). Planar scans were acquired with the patient supine at 10 min, 60 min, 160 min, 20 h, and 24 h after injection. A CT scan was acquired at 95 min, followed by local tomographic SPECT imaging.

**Results:**

One patient was scanned twice with RAD201, 3 months apart, resulting in a total of seven scans for six patients. Here, we show that the use of RAD201 in our patient group shows the same favorable biodistribution as in a previous study with RAD201 (NCT04040686) and that the radiation dose to the critical organ kidney can be reduced by the application of the plasma expander Gelofusine® by almost 50%.

**Conclusion:**

RAD201 appears safe for use in humans and is a promising noninvasive tool for discriminating HER2 status in metastatic (breast) cancer, regardless of ongoing HER2-targeted antibody treatment.

**Supplementary Information:**

The online version contains supplementary material available at 10.1007/s00259-022-06066-3.

## Introduction

The human epidermal growth factor receptor (HER2, ErbB2) is one of four known members of the epidermal growth factor receptor family. HER2 gene (ERBB2) amplification leads to HER2 protein overexpression, activating a variety of signaling pathways leading to cellular proliferation and tumorigenesis [1, 2]. Although the majority of studies on HER2-targeted therapies have focused on breast cancer, HER2 positivity has also been observed in various other cancers, such as those arising in the gastrointestinal tract, urinary bladder, salivary glands, lung, ovaries, colon, and pancreas [3, 4].

Currently, HER2 status is determined ex vivo on biopsy or pathological specimens using several different methods: immunohistochemical assessment (IHC), enzyme-linked immunosorbent assay (ELISA) of serum or tumor cytosol, and Western blot test for overexpression of HER2 protein. In addition, fluorescence in situ hybridization, chromogenic in situ hybridization, silver in situ hybridization, Southern blot, and PCR are used to assess HER2 gene amplification [2]. Despite multiple revisions to the interpretation guidelines and multiple methods developed for HER2 status determination, inaccurate HER2 test results continue to pose a challenge in the treatment of breast cancer patients [5, 6]. Since HER2 serves as a therapeutic target, it is of utmost importance to avoid misclassification in order to give patients the opportunity to receive the most effective therapy for their disease.

In recent years, several groups evaluated the use of radiolabeled single-domain antibodies (sdAb), also called nanobodies. These are the antigen-binding domains of heavy chain-only camelid antibodies. Their small size, low immunogenicity, rapid blood clearance rate, and high affinity for their antigen make them ideal for use in radioimmunoimaging [7, 8]. Despite the small size of the nanobodies facilitating rapid excretion by the glomeruli of the kidneys, retention also occurs in the negatively charged lumens of the tubular system. In the kidney, the endocytic receptors megalin and cubulin, expressed on the apical side of the proximal tubule cells, are responsible for the reabsorption of proteins. The receptors bind and internalize a variety of ligands, posing a major dosimetric problem. This may amount to renal toxicity in a dose-dependent manner, particularly in targeted endoradiotherapy with long-lived radioisotopes, often limiting their applicability [9, 10]. Previous studies have shown that infusion of positively charged amino acids or succinylated gelatin (Gelofusine®) reduced renal retention of peptides and small proteins. Gelofusine® consists of succinylated bovine gelatin molecules and is used clinically as a plasma expander. In the past, infusion of Gelofusine® has been shown to result in increased excretion of megalin ligands, leading to the competitive displacement of radiolabeled proteins and thus decreased renal retention [11]. However, this principle does not apply to all compounds, and efficiencies can vary widely between different types of radiolabeled compounds [12].

Previously, the safety, favorable biodistribution, and imaging characteristics of the technetium-99^m^-labelled sdAb NM-02, now called RAD201, were demonstrated in 10 patients [13]. In this paper, we report on the biodistribution, dosimetry, and tumor targeting potential of this sdAb, with the additional injection of the plasma volume expander Gelofusine® for radiation protection, in heavily pretreated patients with different types of cancer. Observing the same favorable biodistribution (NCT04040686) would make the tracer a promising noninvasive tool to discriminate HER2 status in metastatic (breast) cancer, independent of ongoing HER2-targeted antibody treatment.

## Materials and methods

### Radiopharmaceutical preparation

The synthesis of RAD201 was described in detail before [13, 14]. Briefly, 200 µg NM-02 sdAb was radiolabeled with [^99m^Tc(OH_2_)_3_(CO)_3_]^+^ complex binding to its C-terminal hexahistidine tag [15]. The mixture was incubated at 50 °C for 1 h and then diluted with saline. The mixture was then passed through a 0.22-µm syringe filter into a pyrogen-free, evacuated vial, and quality control was performed by HPLC, TLC, and endotoxin analysis by the Limulus amebocyte lysate assay. In addition, a retrospective sterility test was performed for every batch. The average radiochemical purity of RAD201 was 98.6% (95.6–99.9%); purification after radiolabeling was not necessary. The final injection solution was colorless, with neutral pH and an endotoxin level below 0.2 EU/mL.

### The patients

In 2021, we have clinically introduced RAD201 as an additional diagnostic tool for the evaluation of in vivo HER2-positivity in patients with various primary tumors as single patient use. All patients have been referred for additional examination, informed about the individual medical decision of this new diagnostic procedure and about possible risks and side effects, and signed a written informed consent form. All reported investigations were conducted in accordance with § 13 (2b) German Medicinal Products Act (AMG) [16] and to § 83 (3) German Radiation Protection Act (StrlSchG) [17] as well as the updated Declaration of Helsinki, § 37 (unproven interventions in clinical practice), which includes the priority of approved procedures. Patients were informed by a nuclear medical specialist about the entire imaging procedure, and only after obtaining voluntary informed consent for the imaging procedure were injected with RAD201, as outlined below. A retrospective evaluation was approved by the institutional review board of the local ethics committee at the local medical faculty (retrospective study CTC-A 22–057). In total, six HER2-positive cancer patients aged between 42 and 69 have been examined. The HER2 status of primary tumors and/or metastases was determined by IHC on biopsy samples. The included patients already received several lines of therapies, including HER2-directed therapies, and yet showed progression or suspected recurrence under their respective therapy at the time of RAD201 imaging (Table 1), therefore the additional assessment of current HER2 was deemed important for the individual clinical management. In six of seven scans, patients were administered 500 mL of Gelofusine® solution (40 mg/mL) for radiation protection prior to tracer injection. Patients were asked to void their bladder before injection of 434 ± 42 MBq RAD201, corresponding to 100 μg of NM-02. To monitor potential drug-related adverse events, participating patients had a telephone follow-up 1 week after RAD201 imaging.

**Table 1:** Patient characteristics; time since initial diagnosis is related to the date of the RAD201 SPECT scan

Patient no	Age (y)	Cancer type	Initial TNM	Time since initial diagnosis [months]	HER2 status (primary tumor)	Surgery	Chemotherapy	Radiation therapy	Metastatic lesion (during course of disease)	HER2 status (etastases)
1^*^	53	BC; IDC; ILC	pT2pN0M0	104/107	1 + /0 (r/l)	Yes	Yes	Yes	Bone and lymphogenic	3 +
2	42	BC; NST	cT2cN1cM0	28	3 +	Yes	Yes	Yes	Cerebral and lymphogenic	3 +
3	54	BC; NST	cT2cN1M1	19	3 +	No	Yes	Yes	Bone and hepatic	3 +
4	69	CUP	cTxN2M1	11	-	No	Yes	No	Bone and lymphogenic	3 +
5	59	EC	cT4cN1cM1	2	3 +	No	Yes	Yes	Bone, cerebral, hepatic, and adrenal	3 +
6	56	BC; RC	pT2pN1cM0	142	0	Yes	Yes	Yes	Bone, bone marrow, hepatic, and cerebral	2 +

* = Patient was first scanned without Gelofusine® and 3 months after use of trastuzumab emtansine (T-DM1) with Gelofusine®; HER2 status of patients was determined using IHC; *BC*, breast cancer; *IDC*, invasive ductal carcinoma; *ILC*, invasive lobular carcinoma; *NST*, no special type; *CUP*, carcinoma of unknown primary; *EC*, esophageal cancer; *RC*, rectal cancer

### SPECT/CT scan protocol

All images were obtained using a Siemens Symbia™ T16 SPECT/CT system. Planar scans were acquired with the patient supine at 10 min, 60 min, 160 min, 20 h, and 24 h after injection at 10 cm/slice/min. A low-dose/CT scan was acquired at 95 min, followed by local tomographic SPECT imaging. All scans were acquired using a low-energy high-resolution collimator in a 20% energy window centered around 140 keV, in a 256 × 1024 matrix for planar images and 128 × 128 matrices for tomographic images. A 15% energy window centered around 140 keV was also collected during tomographic acquisitions for attenuation and scatter correction. SPECT images were acquired over 360° in 60 frames per full rotation, with 60-s acquisition per frame.

A calibration source of 20 MBq at injection time was placed next to the patient to provide quantitative calibration of counts to activity. Following CT-based target structure segmentation performed on HERMES GoldLx (V2.11.0.1; Hermes Medical Solutions), dosimetry for relevant organs was analyzed using the Medical Internal Radiation Dose (MIRD) system via OLINDA (V1.1; patient/organ-specific S-values) and ULMDOS (V1.4; residence time calculation). The tumor-targeting potential was assessed in primary and metastatic lesions. Focal RAD201 uptake that was above the background and corresponded to a lesion identified by PET/CT imaging was defined as a positive imaging result. Negative RAD201 imaging was defined as no distinct tracer uptake in lesions previously identified as part of the standard of care.

## Results

Six patients (5 women; mean age, 55.5 years) with histopathologically proven HER2-positive tumors were included. One patient was scanned twice with RAD201 3 months apart, thus a total of seven scans were obtained in six patients. Of the six patients who participated in HER2 imaging with RAD201, dosimetry was performed in only five patients. Since one of the five patients was scanned twice with RAD201, a total of six sets of dosimetry data were acquired, one without and five with Gelofusine® administration prior to tracer injection. The patients received on average 434 ± 42 MBq of RAD201. Patient and study drug characteristics are summarized in Table 1. No signs or symptoms of drug-related adverse effects were reported during the imaging procedure or afterward (telephone interview 1 week after the scan).

**Fig. 1:**
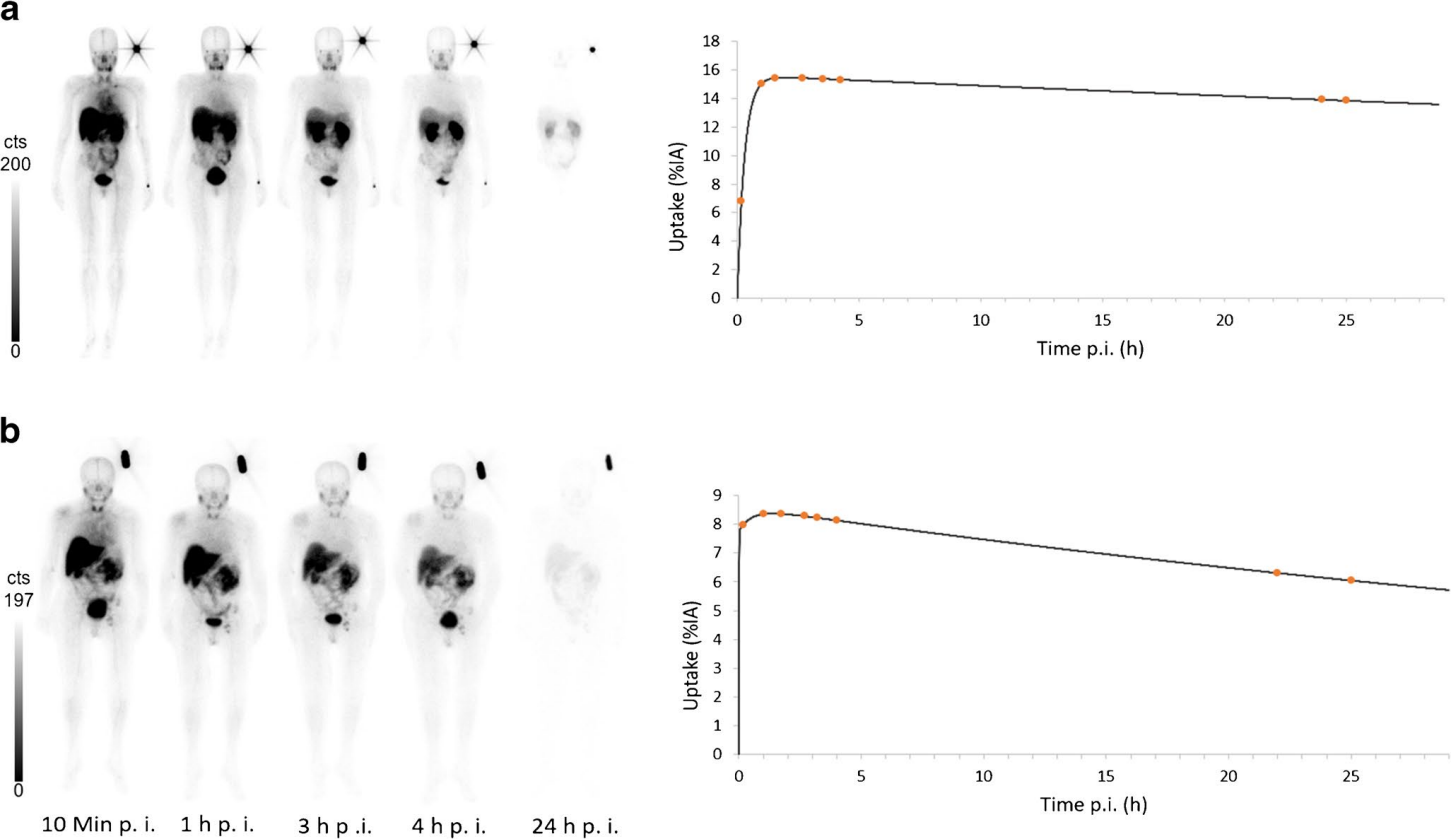
Anterior whole-body images and time-activity curves of the kidney of patient 1a **a** injected only with RAD201 and patient 4 injected with RAD201 and Gelofusine® **b** after 10 min, 1 h, 3 h, 4 h, and 24 h after injection

**Fig. 2:**
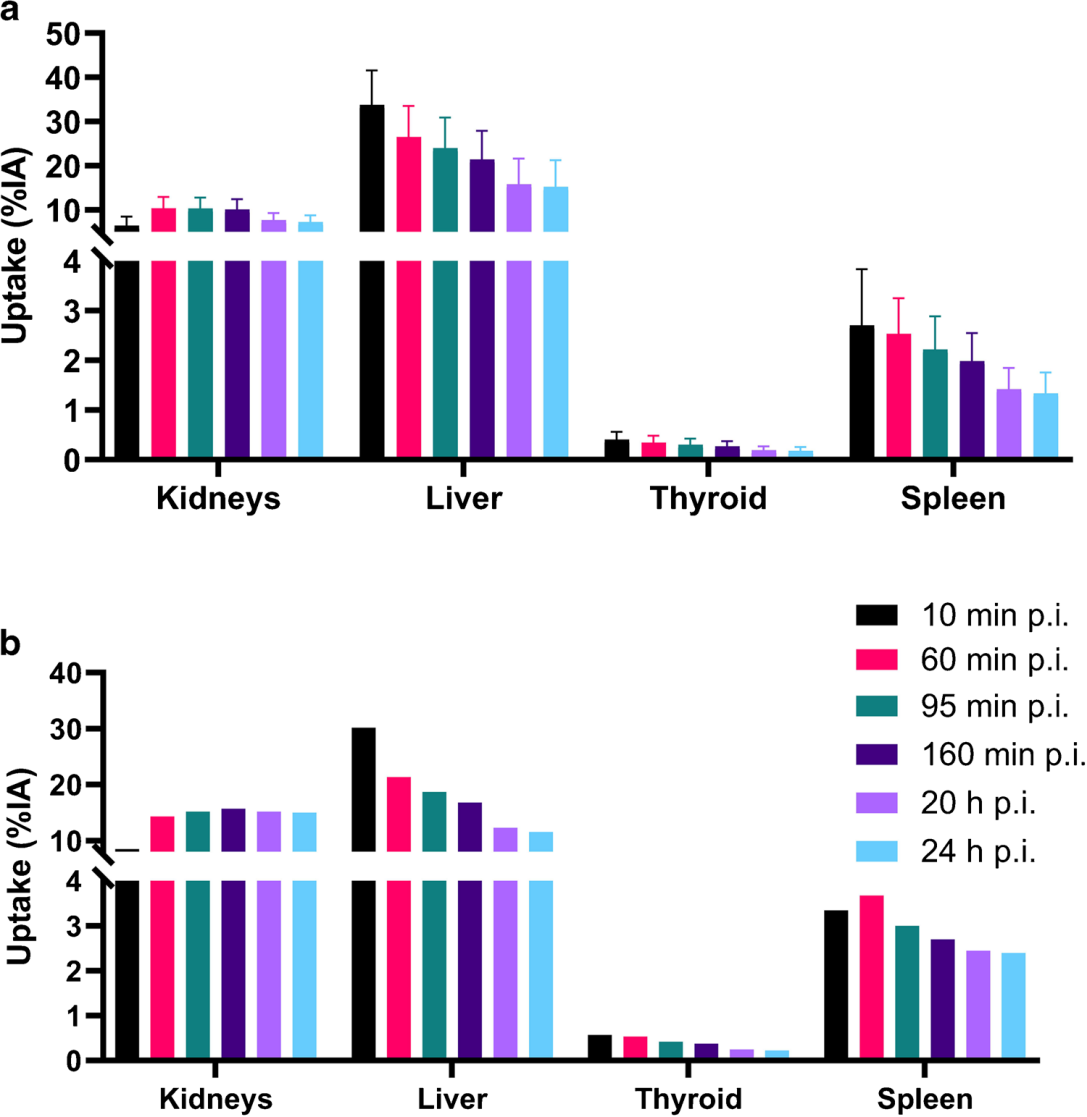
Uptake expressed in percentage injected activity (IA), in different organs for patients injected with **a** Gelofusine® and RAD201 (*n* = 5) and **b** the one patient injected with RAD201 without Gelofusine® (*n* = 1) at 10 min, 60 min, 95 min, 3 h, 20 h, and 24 h post injection

Figure 1 shows the whole-body images of representative patients at different time points after administration of RAD201, with and without Gelofusine®. The uptake of RAD201 in the organs with the highest activity, mainly the kidneys and liver, is shown in Fig. 2. The radiation absorbed dose in the remaining organs was similar to those results published by Zhao et al. [13], except in the thyroids. This uptake pattern was already present in the 10-min images and decreased over time. The high renal activity sustained over 24 h, as well as the continuous excretion into the bladder during the studied time period, indicated retention in the parenchyma as well as renal elimination of the tracer.

**Fig. 3:**
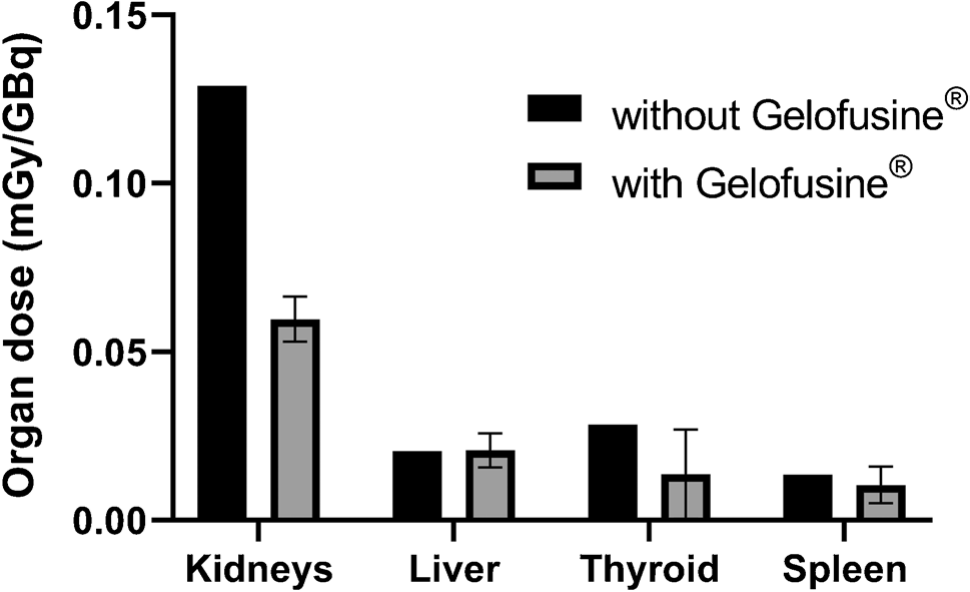
Organ doses for the organs with the highest radiotracer uptake with (*n* = 5) and without Gelofusine.® (*n* = 1)

Figure 3 and Supplemental Table 1 summarize individual organ doses for the six scans of the five patients. All patients had normal liver and kidney functions. The kidneys showed the highest organ dose with and without Gelofusine® (0.06 ± 0.007 mSv/MBq and 0.129 mSv/MBq, respectively) in all patients. In the patients without Gelofusine® injection, the organ dose to the thyroid was 0.028 mSv/MBq and to the liver 0.021 mSv/MBq. There was less retention in the thyroids in the patients who received Gelofusine®, resulting in a lower dose (0.014 ± 0.013 mSv/MBq), while the liver dose (0.021 ± 0.005 mSv/MBq) remained unchanged when compared to the patient who did not receive Gelofusine®. This can be explained by the faster renal elimination and hence lower amount of free technetium after degradation in the liver.

**Fig. 4:**
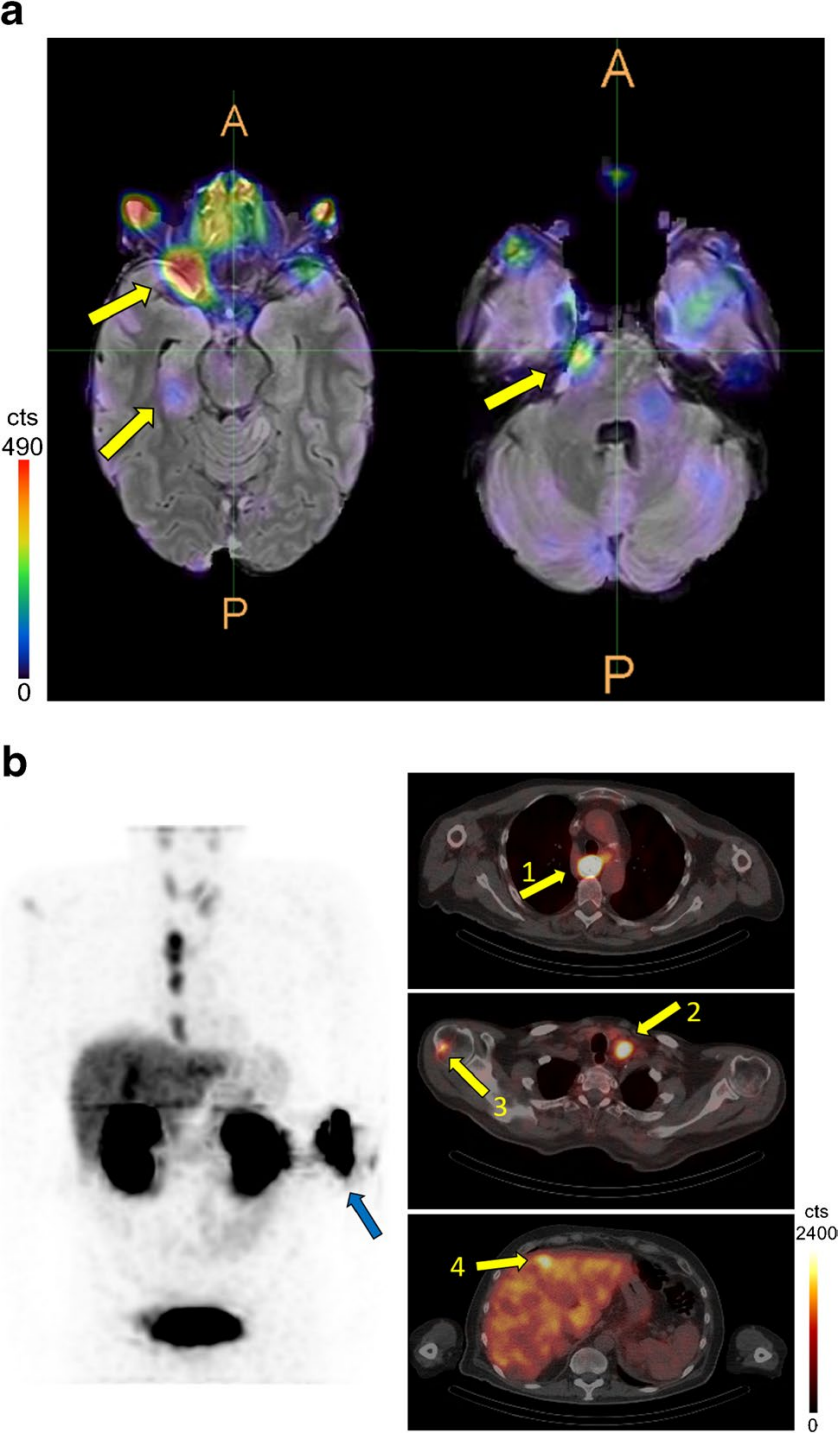
Uptake of RAD201 in **a** brain metastases (arrows) fused with individual MRI images from patient 2. **b** Patient 5 with esophageal cancer (**1**) and metastases in lymph node (**2**), bone (**3**), and liver (**4**). The blue arrow indicates the injection site

Uptake in tumor lesions could be observed in all patients with vital lesions. The number and localization of tumor lesions, as well as the corresponding tumor-to-background (T/B) ratio, are shown in Table 2. All patients had local or distant metastases. Only patient 5 also had a primary lesion at the time of the scans (Table 1, Supporting information). Clear tracer uptake was seen in the vital metastatic lesions in all patients and also in the primary lesion in patient 5. Figure 4 shows RAD201 SPECT/MR images of patient 2 with metastases in the brain and patient 5 with a primary lesion in the esophagus and metastases in the liver.

**Table 2:** Comparison of conventional diagnostic methods with RAD201 SPECT. Because of the general nature of retrospective evaluations of examinations based on the individual medical decision, the patient’s medical histories differ significantly, limiting comparisons on a patient-wide basis. Depending on the pathology and progression of the disease, different imaging modalities were employed for diagnosis. Due to this, it is not possible to determine the number of lesions for all patients. For the SUVmax and tumor-to-background ratio (T/B) ratio, the uptake in the lesion with the highest uptake was measured in the case of several lesions. The same tissue type without recognizable tracer uptake was used as background. The number of lesions detected by conventional diagnostic approaches could not be given for all patients because, in some cases, conventional diagnostics were used only to localize the tumor. All patients were scanned during ongoing HER2-targeted therapy

Patient no.	CD			Time between CD and RAD201 SPECT	RAD201 SPECT	
	Method	Localization of lesions	SUVmax		No. of lesions SPECT/CD	T/B
1a	FDG	Bone	9.69	2 weeks	1/1	3.53
		Lymphogenic	7.2		4/4	2.09
1b	FDG	Bone	2.53	1 week	1/1	1.63
2	MRI	Cerebral	n.a.	7 weeks	3/9	4.94
3	BSc	Bone	n.a.	11 weeks	0/1	-
4	BSc	Bone	n.a.	22 weeks	6/n.a.	5.36
		Lymphogenic			10/n.a.	4.59
5	CT and MRI	Esophagal	n.a.	14 weeks	n.a.	20.44
		Lymphogenic				18.89
		Hepatic				3.11
		Adrenal				7.57
		Bone				18.33
6	FDG	Bone marrow	6.23	9 weeks	1/1	5.25
		Hepatic	9.12		0/1	-

*CD*, conventional diagnostics; *FDG*, ^18^F-fluorodeoxyglucose; *MRI*, magnetic resonance imaging; *BSc*, bone scintigraphy; *T/B*, tumor to background; *n.a.*, not applicable

**Fig. 5:**
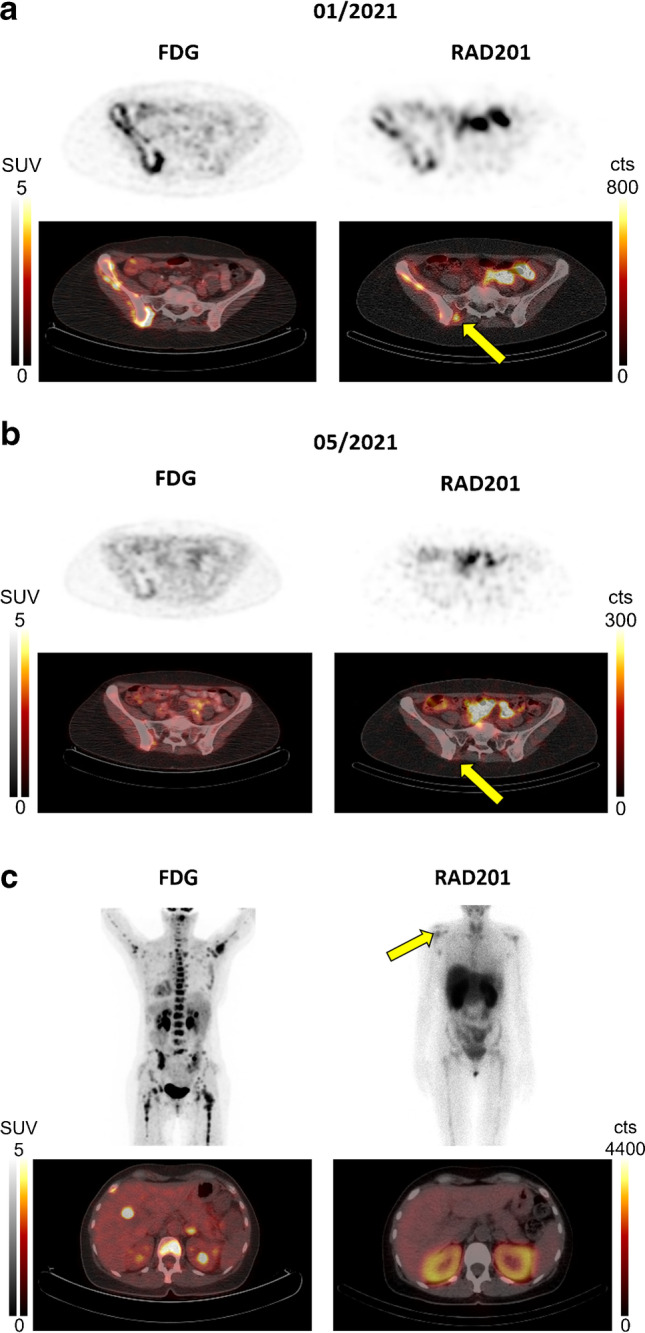
**a** The first ^18^F-FDG PET images of patient 1 were obtained 2 weeks before the RAD201 SPECT and showed a metabolic active metastasis in the right os ilium with a somewhat heterogeneous uptake of RAD201 in the same location (arrow). **b** After 3 months of therapy, an ^18^F-FDG scan and RAD201 scan were performed again, which revealed an almost vanished uptake in this metastasis. **c** ^18^F-FDG PET images of patient 6 were obtained 3 months before the RAD201 SPECT and showed a tracer uptake in the liver and bone marrow. RAD201 Scan only showed uptake in the bone marrow with accentuations in both shoulders

RAD201 tracer accumulation observed in patient 1 matched well with the uptake patterns observed in a ^18^F-fluorodeoxyglucose (FDG) scan acquired a few weeks beforehand (Fig. 5a and b). To monitor the progress of the HER2-targeting therapy T-DM1 (trastuzumab emtansine), FDG and RAD201 scans were performed 3 months after the first RAD201 scan. Again, the FDG and RAD201 scans matched well (Table 2) and showed an almost complete response to therapy, a reduction in tumor mass (all lymph node metastases were no longer detectable), and an almost complete reduction of uptake in the bone metastases in the os ilium.

An important finding in this set of RAD201 SPECT/CT scans is the possibility of detecting intratumoral heterogeneity of HER2 expression, as shown in patient 6 (Fig. 5c). While the FDG scan showed a solitary liver metastasis and diffuse bone marrow involvement, RAD201-imaging suggested that only the bone marrow carcinomatosis affecting the shoulders was HER2 positive.

## Discussion

We have observed that RAD201 SPECT/CT imaging in the setting of a single patient use (as described in AMG) appears to be a safe procedure, and the tracer administration causes no observable adverse reactions or serious adverse reactions. RAD201 showed a favorable biodistribution, with the highest uptake in the kidneys, liver, thyroid, and spleen but very low background levels in all other organs, which was also observed in a previous study on this radiotracer [13].

The rapid excretion of RAD201 from the blood pool via the kidneys led to a rapid reduction in nonspecific background activity in the blood pool and organs, allowing SPECT imaging as early as 2 h after injection. The increased activity in the kidneys and bladder suggests that RAD201, like many other nanobodies, is mainly excreted renally [14, 18–21].

While preclinical studies previously demonstrated a 36% reduction in renal activity by co-injection of Gelofusine® with the nanobody ^99m^Tc-7C12 and a reduction of 95% for ^177^Lu-DTPA-untagged-2Rs15d [19, 21], the current data are the first to indicate a 46% reduction in renal activity in clinical use with radiolabeled sdAb tracers. A 50% reduction in uptake in the thyroids can also be observed by the application of Gelofusine®; this is most likely explained by less free technetium due to faster clearance; however, the number of patients who did not receive Gelofusine® is too small for a precise explanation. The high standard deviation in the thyroid may arise from the different amounts of free technetium pertechnetate contained in the product, although the percentage was always less than 5%.

In patient 1, two ^18^F-fluorodeoxyglucose (FDG) and RAD201 scans were performed within 3 months to first determine the HER2 status of the metastasis and subsequently evaluate the course of a newly initiated therapy. This showed that the FDG scans matched well with the RAD201 scans, with the latter allowing the tumor to be more accurately localized and delineated from healthy tissue. The difference in uptake in the tumor region before and after treatment shows a clear therapy effect and underlines the assumption that RAD201 can be used to evaluate treatment efficiency.

While a previous study successfully demonstrated that RAD201 has acceptable radiation dosimetry with favorable biodistribution characteristics and has the potential to image untreated primary tumors [13], our observational data additionally demonstrated optimal biodistribution and high tumor accumulation even in heavily pretreated patients with late-stage metastatic disease. This is mainly due to the fact that the NM-02 sdAb, the core of RAD201, binds to HER2 on a different epitope than the most common HER2-targeting immunotherapeutic, trastuzumab. In all six patients, the application of Gelofusine® appears to improve biodistribution resulting in a reduction of renal radiation exposure compared to an already published cohort of patients [13] and additionally, the observation in one patient intraindividual. Importantly, when comparing RAD201 biodistribution in patients from a previous study [13] and those presented here, the plasma expander seems to have no impact on RAD201 tumor accumulation.

A direct comparison between conventional diagnostics and RAD201 imaging is not possible for this retrospective analysis because different methods are used to diagnose patients depending on their pathology and progression of the disease. In addition, in some cases, there is a larger time gap between conventional imaging and the RAD201 imaging during which the patients were under treatment. Nevertheless, most of the lesions detected by conventional diagnostics could also be localized by RAD201 imaging. The largest discrepancy in the number of detected lesions was found in patient 2. While nine lesions were detected in the MRI images of the brain, only three were detected in the RAD201 imaging. However, this could also be due to the whole-head irradiation that the patient received in the meantime (Supplemental information). In patient 3, the tracer enhancement observed in the bone scintigraphy could not be observed in the RAD201 imaging. However, since the question in this patient was to clarify the tumor’s viability, it can be concluded that no active metastases were observed under treatment. This assumption can be confirmed by another bone scintigraphy, 1 year after RAD201 imaging, in which no changes in bone metastases’ configuration and distribution were observed.

Despite the promising results, there are some limitations in this retrospective study. First, the cohort in the two groups of interest (with and without Gelofusine®) was too small. For a meaningful comparison and statistical analysis, RAD201 should be investigated in a large-scale clinical study. Second, although we could detect the tracer accumulation in different metastases, only three of the six patients had another primary cancer than breast cancer. Finally, all patients had at least one histologically proven HER2-positive lesion. Therefore, in some patients, the true-positive results could be assessed but not the true-negative rate. However, this has already been investigated in a small cohort [13].

We could also demonstrate that metastases of a tumor entity other than breast cancer, i.e., HER2-positive esophageal cancer, could be delineated by RAD201 SPECT/CT imaging. Furthermore, we can conclude that RAD201 imaging has the potential to be applied for the detection of the liver, lymph node, and bone metastases, as well as intracranial metastases. The latter aspect supports the notion that this tracer is most likely crossing the blood–brain barrier and could be a valuable tool for the detection as well as treatment of intracranial metastases.

## Conclusion

We demonstrated that RAD201 has a favorable biodistribution, with a high target-to-background ratio and a high level of accumulation at all active HER2-positive tumor sites, including visceral foci, lymph nodes, and skeletal and intracranial lesions. We showed that by using a plasma expander, the radiation dose to the critical organ (kidneys) seems to be reduced by almost 50% without affecting uptake in tumor sites. RAD201 demonstrated favorable tumor targeting and rapid blood clearance despite ongoing HER2-targeted therapy, allowing SPECT/CT imaging within a few hours after injection. These characteristics of RAD201 warrant further evaluation in a prospective clinical setting.

## Supplementary Information

Below is the link to the electronic supplementary material.

Supplementary file1 (DOCX 46 KB)

## Data Availability

The datasets generated and analyzed during the medical examination are available from the corresponding author upon reasonable request.

## Materials availability

The datasets generated and analyzed during the medical examination are available from the corresponding author upon reasonable request.

## Abbreviations

BC: Breast cancer
BSc: Bone scintigraphy
CUP: Carcinoma of unknown primary
EC: Esophageal cancer
HER2: Human epidermal growth factor receptor 2
IDC: Invasive ductal carcinoma
IHC: Immunohistochemistry
ILC: Invasive lobular carcinoma
ELISA: Enzyme-linked immunosorbent analysis
NST: No special type
sdAb: Single domain antibody
T-DM1: Trastuzumab emtansine
