# Supplementary material for: 99mTc‑labeled single-domain antibody for SPECT/CT assessment of HER2 expression in diverse cancer types
Source: Eur J Nucl Med Mol Imaging. 2022 Dec 9;50(4):1005–13. doi: 10.1007/s00259-022-06066-3 (PMC9931776; doi:10.1007/s00259-022-06066-3)
Supplement: Supplementary file 1 — Supplementary file1 (DOCX 46 KB) [file 259_2022_6066_MOESM1_ESM.docx]

**Supplementary information**

**Patient information**

Case 1. The first patient was a 53-year-old woman, first diagnosed in June 2012 with invasive ductal carcinoma (IDC) of the right breast (estrogen receptor (ER)^+^ and progesteron receptor (PR)^+^, HER2^-^ (1+), ypT2, pN0) and invasive lobular breast carcinoma on the left (ER^+^ and PR^+^, HER2^-^ (0), ypT0, pN0). The patient was initially treated with six cycles of TAC (Taxan, Anthrazyklin, Cyclophosphamid). In December 2012, a mastectomy and axillary dissection was performed on the right breast and a breast conservation therapy (BCT) was performed on the left breast. The patient also received standard adjuvant radiotherapy after surgery and adjuvant tamoxifen. In February 2014, several osseous metastases were detected in the pelvis and the pelvic and lumbar spines, as well as axillary and infraclavicular lymph node metastases on the right thoracic wall (G2, ER^+^, PR^-^, HER2^+^). After radiotherapy of the right acetabulum, therapy with denosumab was started and supplemented after one month with HER2-directed therapy (trastuzumab/pertuzumab) and taxol. The immunotherapy regimen had to be changed several times due to various side effects and moderate heart failure, it was switched to zoledronic acid, trastuzumab, pertuzumab and letrozole in June 2018. In January 2021, therapy was switched to T-DM1.

RAD201 imaging was applied to this patient to track the therapy efficiency.

Case 2. A 42-year-old woman was diagnosed with right breast cancer (ER^+^, PR^-^, HER2^+^, cT2, cN1, cM0) during pregnancy in November 2018. Prior to delivery, neoadjuvant chemotherapy with epirubicin and cyclophosphamide was administered. After the birth in January 2019, paclitaxel and trastuzumab/pertuzumab were additionally administered. After a segmental resection of the right breast in June 2019, adjuvant radiotherapy was administered and therapy was continued with trastuzumab/pertuzumab, as well as goserelin and tamoxifen. After cerebral metastasis (ER+, HER2^+^) was diagnosed in September 2020, resection followed by adjuvant radiotherapy was performed. During an MRI scan in January 2021, meningiosis neoplastica was suspected, which was determined to be meningiosis carcinomatosa by performing a lumbar puncture and CSF sampling. The therapy was then changed to T-DM1 with Zoladex. However, as a progression was observed after two cycles, whole head irradiation was performed in March 2021.

RAD201 imaging was applied to determine whether all metastases are HER2 positive.

Case 3. In November 2019, a 54-year-old premenopausal woman was diagnosed with right breast cancer (NST, ER^+^, PR^-^, HER2^+^ (3+), cT2, cN1, M1,) with metastases in the thoracic vertebral body and liver. HER2 directed therapy with trastuzumab/pertuzumab, paclitaxel and denosumab was started first, followed by palliative radiotherapy. In parallel to the radiotherapy, a systemic therapy with carboplatin was applied. After completion of the radiotherapy, the therapy was continued with trastuzumab/pertuzumab, letrozole and ibandronate.

RAD201 imaging was applied to check the vitality of the metastases.

Case 4. In September 2020, a 69-year-old female patient was diagnosed with adeno carcinoma of unknown primary origin (cTx, N2, M1). In addition, a Paget´s carcinoma of the vulva with soft tissue metastasis infiltrating the perineal muscles, polytopic osseous metastases, and a right inguinal, iliac, retroperitoneal lymph node metastasis were detected. The receptor status of the lymph node metastasis was determined to be ER^-^, PR^-^ and HER2^+^ (3+). As therapy, paclitaxel, dual blockade and denosumab administration was started.

RAD201 imaging was performed to restage the patient and to evaluate whether a primary tumor could be delineated.

Case 5. A 59-year-old male patient was diagnosed with stage 4 esophageal cancer AEG type 1 in June 2021 (cT4, cN1, cM1). In addition, the patient had HER2^+^ (3+) hepatic, osseous, adrenal, and multiple cerebral metastases. For therapy, the patient received two cycles of FOLFOX plus trastuzumab and whole brain irradiation. In addition, radiation to the right metatarsal was performed to treat the osseous metastasis.

RAD201 imaging was performed for therapy evaluation of HER2-positive tumor, because of inconsistent diagnostic findings, CT progress, bone scan stable disease. No HER2-positive/vital tumor was detected which was confirmed by a completely unchanged follow up bone scan one year later.

Case 6. The sixth patient was a 56-year old woman first diagnosed in 2010 with left breast cancer (ER^+^, PR^+^, HER2^-^, pT2, pN1, cM0, V0, G3, R0), initially treated with lumpectomy and antihormonal treatment. Five years later, the patient relapsed and was diagnosed with recurrent breast cancer, this time HER2 slightly positive (ER^+^, PR^-^, HER2^+^ (2+), rpT1c, pNx, L0, V0, G3, R0), and mastectomy of the left breast was performed. In 2018 a second tumor (rectal carcinoma) was diagnosed and treated with radiotherapy. Following diagnosis of osseous metastases in 2021, in December 2021 a disseminated bone marrow carcinosis and hepatic metastasis were detected. In addition, during a MRI scan in December 2021, meningiosis carcinomatosa and cerebral metastasis were suspected. As therapy, cerebral irradiation and polychemotherapy were started beginning of 2022. Imaging was performed to disentangle the option of potential targeted therapy towards HER2 positive metastases.

**Supplemental Table 1**: Organ doses. No dosimetry was performed for patient no. 6. The mean ± SD include only patients that received Gelofusine^®^.
